# Age-Related Cataract Is Associated with Elevated Serum Immunoglobulin E Levels in the South Korean Population: A Cross-Sectional Study

**DOI:** 10.1371/journal.pone.0166331

**Published:** 2016-11-18

**Authors:** Tae Keun Yoo, Sun Woong Kim, Kyoung Yul Seo

**Affiliations:** 1 Institute of Vision Research, Department of Ophthalmology, Yonsei University College of Medicine, Seoul, South Korea; 2 Department of Ophthalmology, Yonsei University Wonju College of Medicine, Wonju, South Korea; Soochow University Medical College, CHINA

## Abstract

**Background:**

Previous research has suggested that immunoglobulin E (IgE)-mediated events lead to several chronic diseases. We investigated the association between allergic conditions and age-related cataracts in the South Korean adult population.

**Methods:**

A cross-sectional study was performed using data obtained from 1,170 participants aged 40 years or older who were enrolled in the Korean National Health and Nutrition Examination Survey 2010. Multivariable logistic regression was used to examine the relationship between age-related cataracts and allergic conditions, including total serum IgE and allergen-specific serum IgE levels, after adjustment for potential confounders (age, sex, alcohol consumption, smoking, sun exposure, blood pressure, plasma glucose and cholesterol levels, as well as histories of asthma, atopic dermatitis, and rheumatoid arthritis).

**Results:**

After adjusting for potential confounders, the odds ratio (OR) for age-related cataract was greater in participants with higher total serum IgE levels (OR = 1.37; *P* = 0.044). In particular, increased IgE levels were significantly associated with nuclear cataract (OR = 1.42; *P* = 0.032). However, allergen-specific serum IgE levels did not differ significantly between groups. In the trend analysis, no significant relationship was observed between serum IgE and any type of age-related cataract.

**Conclusion:**

Increased total serum IgE level is independently associated with age-related cataracts after adjustment for confounding factors.

## Introduction

Cataract, defined as opacity of the crystalline lens, is the most common cause of visual loss worldwide [1]. Moreover, the prevalence of age-related cataract will increase rapidly as society ages; this will produce a huge socioeconomic burden worldwide. Nonetheless, the exact mechanisms that lead to age-related cataract remain unclear. In recent years, research has focused on the systemic conditions that increase the risk of age-related cataract. In particular, glycation and oxidative change of lens proteins as a result of elevated glucose levels in serum and aqueous humor has been recognized as a major pathophysiological process in cataract formation [2]. Systemic oxidative stress is also a risk factor [3]. Conversely, several population-based studies have indicated that various antioxidants prevent cataract development [4]. Additionally, increased prevalence of cataract has been reported in patients with history of smoking, hypertension, steroid use, and celiac disease [5,6]. Socioecomomic status was also associated with age-related cataract [7].

Immunoglobulin E (IgE) is an antibody that binds to Fc (fragment, crystallizable) receptors, which are mostly found on the surface of mast cells [8]. It plays a key role in the signaling response to allergens. The binding of IgE to the Fc receptor activates mast cell degranulation, as well as the release of mediators such as cytokines, chemokines, histamine, proteoglycan, and mast cell protease. These mediators induce a reaction that is typical of hypersensitivity: vascular leakage, vasodilation, and airway constriction [9]. Clinically, increased IgE levels have been found in patients with atopic dermatitis, asthma, and hay fever [10]. In more recent research, increased IgE has also been associated with several chronic diseases, including rheumatoid arthritis [11], atherosclerosis [12], ischemic heart disease [9], and diabetes mellitus [13,14]. In particular, the inflammatory mediators released from mast cells may increase capillary permeability and joint inflammation in patients with rheumatoid arthritis, and they may trigger vasoconstriction and endothelial cell remodeling in patients with atherosclerosis [15,16]. Moreover, a previous report have suggested that these mediators increase cytokine-induced insulin resistance and impair insulin secretion [13].

It is well-known that anterior and posterior subcapsular cataracts are a common ocular complication of atopic dermatitis [17]. However, the association between age-related cataract and allergic conditions remains unclear—although previous studies have suggested that systemic inflammation is involved in the progression of age-related cataract [18,19]. Because the IgE-mediated immune response may be a major trigger of systemic inflammation, we hypothesized that serum IgE level is associated with the development of age-related cataract.

In the present study, we investigated whether increased serum IgE is associated with the prevalence of age-related cataract after adjustment for well-known risk factors (age, sex, body mass index [20], smoking [21], alcohol use [22], sun exposure [23], blood pressure [5], plasma glucose level [2], cholesterol level [24], and history of chronic diseases associated with steroid use [25]).

## Methods

All analyses were based on the Korean National Health and Nutrition Examination 2010 (KNHANES; available online at http://knhanes.cdc.go.kr). The study protocol was approved by the institutional review board of the Korean Center for Disease Control and Prevention (IRB Number: 2010-02CON-21-C). All participants signed forms consenting to the use of their health information in the study. The KNHANES 2010 was a nationwide, population-based, cross-sectional survey conducted by the Division of Chronic Disease Surveillance of the Korea Centers for Disease Control and Prevention [26]. All participants were randomly selected from among 192 surveys conducted at 131 locations using stratified sampling in which the following factors were considered: population, gender, age, regional area, and type of residential area. The KNHANES itself comprised health records that were based on a health interview, a health examination, and a nutrition survey. Specially, the 2010 KNHANES survey gathered information on total and allergen-specific serum IgE [27].

**Fig 1:**
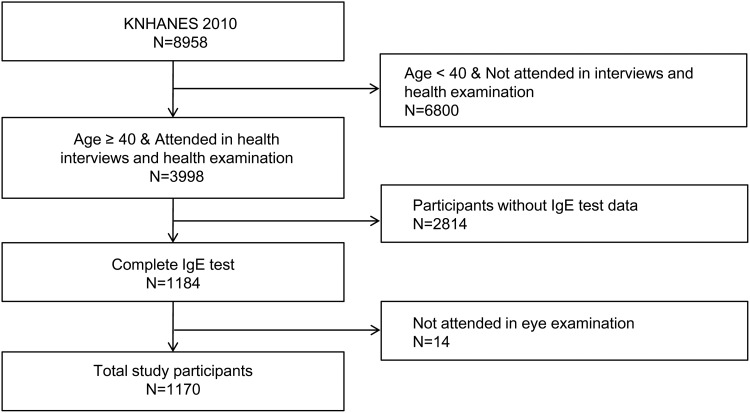
Flowchart of participant selection from the Korean National Health and Nutrition Examination Survey 2010.

A flow diagram of the inclusion and exclusion procedure is shown in Fig 1. A total of 8,958 participants were enrolled in the KNHANES 2010. However, we excluded from the present study (1) those under 40 years of age, in whom age-related cataracts are not a concern, (2) those without either a health interview or examination data (n = 6,800), (3) participants in whom IgE level was not measured (n = 2,814), and (4) subjects who had not undergone an eye examination (n = 14). Ultimately, 1,170 participants were eligible for the current analysis.

A structured eye examination was performed using a slit-lamp (Haag-Streit model BQ-900; Haag-Streit AG, Koeniz, Switzerland) to evaluate the participants’ cataract status [28]. The standard Lens Opacities Classification System III (LOCS III) was used to categorize the type of cataract. Cataracts were compared with standard photographs and classified as cortical (LOCS III score ≥2 for cortical cataracts), nuclear (LOCS III score ≥4 for nuclear opalescence or nuclear color), anterior subcapsular (LOCS III score ≥2 for anterior subcapsular cataracts), posterior subcapsular (LOCS III score ≥2 for posterior subcapsular cataracts), or mixed type (more than one cataract type per eye). Pseudophakic eyes were categorized into the same group as those that had undergone previous cataract surgery. To analyze risk factors associated with each type of age-related cataract, we defined the cataract patients into subtypes. Participants who had a predominantly cortical cataract in at least one eye with no other type of cataract in either eye were defined as having a pure cortical cataract. Participants with a nuclear cataract or an anterior and posterior capsular cataract were defined in a similar manner. Participants who had different cataract subtypes in each eye were defined as having mixed cataract. All ophthalmologic examinations were performed by 154 well-trained ophthalmologists or ophthalmology residents. To reduce inter-observer variation, participating investigators were periodically trained by acting staff members of the National Epidemiologic Survey Committee of the Korean Ophthalmologic Society. All ophthalmic investigators were masked to other data, including serum IgE level. The quality of the survey and cataract evaluation was verified by the Epidemiologic Survey Committee of the Korean Ophthalmologic Society [7].

Demographic information on traditional cataract-related risk factors was obtained during the health interview. Specifically, each participant completed a questionnaire in which they provided information on age, smoking status, alcohol use, sun exposure, and medical history. Blood pressure was also measured by the health professionals. Sunlight exposure time was evaluated by a multiple-choice question with the following answer options: <2 hours, 2–5 hours, and >5 hours per day. Fasting blood samples of individual participants were collected to measure serum biomarkers (fasting glucose, total cholesterol, triglyceride, and serum IgE). The blood samples were immediately refrigerated and transported to the Central Testing Institute in Seoul, South Korea. Levels of total serum IgE and antigen-specific IgE against *Dermatophagoides farinae*, cockroach, and dog allergen were also examined using anImmunoCAP100 kit (Phadia, Uppsala, Sweden) and a 1470 WIZARD gamma-Counter analyzer (PerkinElmer, Turku, Finland) [27]. The upper limits of detectable total IgE and antigen-specific IgE were 5000 kU/L and 100 kU/L, respectively. Total IgE levels of more than 100 kU/L were categorized into the “increased IgE” group, and participants with allergen-specific IgE levels of 0.35 kU/L or more were defined as sensitized [29].

The characteristics of the participants in terms of cataract status were compared using the χ^2^ test (for categorical data) or the Wilcoxon rank-sum test (for continuous data). To assess the association between cataract and increased IgE levels and sensitization to specific antigens, we used multivariable logistic regression to estimate the respective odds ratio (OR). The estimated ORs were calculated in the following ways: crude OR, in which no adjustment was made for potential confounders; Model 1, wherein the data were adjusted for age, sex, body mass index [20], smoking [21], alcohol use [22], and sun exposure [23] (Model 1); Model 2, wherein data were adjusted for all the variables in Model 1 plus factors associated with metabolic and chronic diseases (systolic blood pressure [5], fasting plasma glucose [2], and total cholesterol [24], as well as asthma [25], atopic dermatitis [17], and rheumatoid arthritis [30] where there is a high probability of steroid use). As the 2010 KNHANES did not measure cumulative steroid dose, the adjustment for steroid use was performed using only disease status data for asthma, atopic dermatitis, and rheumatoid arthritis.

To avoid collinearity between the confounders (systolic and diastolic pressure, or total cholesterol and triglyceride), we selected systolic blood pressure and total cholesterol level as covariates. When calculating the correlation matrix of all covariates, there was no multi-collinearity problem (all pairwise Pearson's correlations: *r* < 0.5). We considered *P*-values < 0.05 as statistically significant. All statistical analyses were completed using the SPSS Statistics 21.0 software (IBM SPSS Inc., Chicago, IL, USA).

## Results

**Fig 2:**
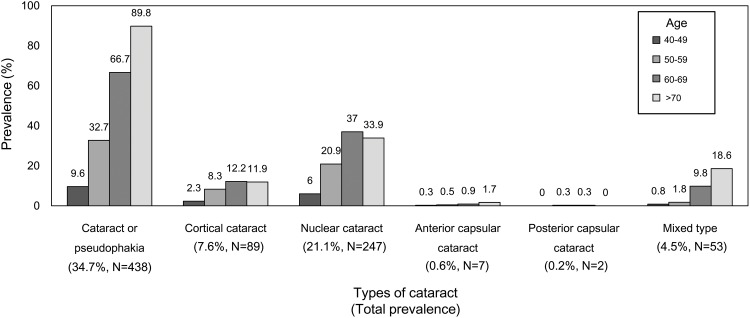
Prevalence of each cataract type within each age group in the Korean National Health and Nutrition Examination Survey 2010.

Ultimately, a total of 1,170 participants were included in this study. In Table 1, the general characteristics of the study participants are arranged according to their cataract status. Participants with age-related cataract were more likely to be older; they also drank alcohol more frequently and had higher systolic blood pressure, lower diastolic blood pressure, higher fasting plasma glucose, and higher IgE levels. Fig 2 presents the prevalence of cataracts in terms of their LOCS status. The prevalence of cataracts or pseudophakia increased as the participants became older. Nuclear cataract was the most common type of cataract in the KNHANES 2010.The total prevalence of cataracts or pseudophakia among the study population was 34.7%. The cataract types and their prevalence rates were as follows: cortical cataract (7.6%), nuclear cataract (21.1%), anterior subcapsular cataract (0.6%), posterior subcapsular cataract (0.2%), and mixed cataract (4.5%). Table 2 shows the number of patients affected by each cataract type in each serum IgE concentration quartile. Patients in higher serum IgE quartiles had a significantly higher prevalence of nuclear cataract than those in lower serum IgE quartiles (*P*-value for linear trend = 0.018).

**Table 1:** Demographic and clinical characteristics of the study participants.

	Total participants (N = 1170)	Cataract or pseudophakia (N = 438)	No cataract (N = 732)	*P*-value
Age (years)	55.0 ± 9.4	61.6 ± 8.2	51.1 ± 7.8	<0.001
Female (%)	582 (49.7)	255 (51.4)	357 (48.8)	0.398
BMI (kg/m^2^)	24.1 ± 2.9	24.2 ± 3.2	24.0 ± 2.8	0.127
House income				
Very low (%)	247 (21.3)	135 (31.3)	112 (15.4)	< 0.001
Low (%)	293 (25.3)	112 (26.0)	181 (24.9)	
Moderate (%)	279 (24.1)	92 (21.3)	187 (25.8)	
High (%)	338 (29.2)	92 (21.3)	246 (33.9)	
Education				
≤ Middle school	532 (46.0)	274 (63.0)	258 (35.7)	< 0.001
≥ High school	625 (54.0)	161 (37.0)	464 (64.3)	
Region of residence				
Urban (%)	898 (76.8)	324 (74.0)	574 (78.4)	0.086
Rural (%)	272 (23.2)	114 (26.0)	158 (21.6)	
Smoking				
No smoking (%)	629 (53.8)	242 (55.3)	387 (52.9)	0.066
Ex-smoking (%)	264 (22.6)	108 (24.7)	156 (21.3)	
Current smoking (%)	277 (23.7)	88 (20.1)	189 (25.8)	
Alcohol use (≥1 drink/week, %)	511 (43.7)	162 (37.0)	349 (47.7)	<0.001
Sun exposure				
<2 hours/day (%)	679 (57.9)	239 (54.6)	440 (60.0)	0.051
2–5 hours/day (%)	299 (25.6)	111 (25.3)	188 (25.7)	
>5 hours/day (%)	192 (16.4)	88 (20.1)	104 (14.2)	
Asthma (%)	48 (4.1)	21 (4.8)	27 (3.7)	0.365
Atopic dermatitis (%)	35 (3.0)	12 (2.7)	23 (3.2)	0.727
Rheumatoid arthritis (%)	40 (3.4)	18 (4.1)	22 (3.0)	0.323
SBP (mmHg)	122.8 ± 17.2	126.39 ± 17.0	120.7 ± 17.0	<0.001
DBP (mmHg)	77.5 ± 10.3	76.7 ± 9.4	77.9 ± 10.8	0.036
Fasting plasma glucose (mg/dL)	101.3 ± 25.0	103.4 ± 24.6	100.1 ± 25.2	0.028
Total cholesterol (mg/dL)	193.7 ± 37.9	191.2 ± 37.5	195.3 ± 38.1	0.075
Triglyceride (mg/dL)	147.8 ± 116.4	146.7 ± 90.1	148.4 ± 129.7	0.797
Increased total serum IgE (%)	550 (47.0)	233 (53.2)	317 (43.3)	<0.001
Sensitization to specific allergen				
*Dermatophagoides farina* (%)	394 (33.7)	152 (34.7)	242 (33.1)	0.566
Cockroaches (%)	236 (20.2)	90 (20.5)	146 (19.9)	0.822
Dogs (%)	51 (4.4)	19 (4.3)	32 (4.4)	0.974

BMI, body mass index; DBP, diastolic blood pressure; IgE, immunoglobulin E; SBP, Systolic blood pressure.

**Table 2:** The prevalence of each cataract subtype in patients with various serum IgE levels.

	Serum IgE quartile (kU/L)				*P*-value for χ^2^ test	*P*-value for linear trend
	<35.0	35.0–37.6	37.7–267.0	>267.0		
Cortical cataract	12	29	22	26	0.038	0.084
Nuclear cataract	51	51	74	71	0.015	0.018
Anterior subcapsular cataract	1	3	3	0	0.740	0.608
Posterior subcapsular cataract	1	0	0	1	0.920	0.998
Mixed cataract	17	11	10	15	0.554	0.655
Pseudophakia	6	13	12	9	0.417	0.567

Table 3 presents the relationship between the various confounding factors and the risk of age-related cataract. Before adjustment, logistic regression showed that age, alcohol use, sun exposure (more than 5 hours per day), systolic blood pressure, diastolic blood pressure, fasting glucose level, and increased serum IgE level were risk factors for age-related cataracts. Increased serum IgE remained a significant risk factor after adjustment in Model 1 (OR = 1.36; *P* = 0.048) and Model 2 (OR = 1.37; *P* = 0.044). In contrast, sensitization to specific antigens, including *Dermatophagoides farinae*, cockroach, and dog, was not significantly associated with age-related cataract either before or after adjustment. The possible effects of socioeconomic status on the association between IgE and age-related cataract were also examined. Additional adjustments for household income and education had no effect on the associations reported above, which showed an age-related cataract OR of 1.41 in Model 1 (*P* = 0.028) and 1.42 in Model 2 (*P* = 0.028) in subjects with an increased IgE level. Therefore, the effect of socioeconomic status on the association between IgE and age-related cataract was minimal.

**Table 3:** Logistic regression analysis of the association between age-related cataracts and demographic or clinical factors.

	Crude		Model 1*		Model 2†	
	OR (95% CI)	*P*-value	OR (95% CI)	*P*-value	OR (95% CI)	*P*-value
Age (years)	1.16 (1.14–1.18)	<0.001	1.16 (1.14–1.18)	<0.001	1.16 (1.14–1.18)	<0.001
Female	1.11 (0.87–1.40)	0.390	1.01 (0.64–1.61)	0.921	1.04 (0.68–1.58)	0.851
BMI (kg/m^2^)	1.03 (0.99–1.07)	0.116	0.99 (0.65–1.26)	0.838	1.00 (0.95–1.05)	0.992
House income						
Very low	1.00 (reference)		1.00 (reference)		1.00 (reference)	
Low	0.51 (0.34–0.72)	< 0.001	1.09 (0.72–1.67)	0.659	1.10 (0.72–1.84)	0.644
Moderate	0.40 (0.28–0.58)	< 0.001	1.47 (0.94–2.31)	0.088	1.49 (0.95–2.35)	0.081
High	0.31 (0.21–0.43)	< 0.001	1.19 (0.76–1.85)	0.428	1.19 (0.76–1.86)	0.425
Education						
≤ Middle school	1.00 (reference)		1.00 (reference)		1.00 (reference)	
≥ High school	0.32 (0.25–0.41)	< 0.001	1.01 (0.72–1.39)	0.968	1.01 (0.72–1.39)	0.975
Region of residence						
Urban	1.00 (reference)		1.00 (reference)		1.00 (reference)	
Rural	1.27 (0.96–1.68)	0.082	1.21 (0.86–1.70)	0.274	1.21 (0.86–1.71)	0.258
Smoking						
No smoking	1.00 (reference)		1.00 (reference)		1.00 (reference)	
Ex-smoking	1.11 (0.82–1.48)	0.496	1.00 (0.61–1.64)	0.987	1.00 (0.61–1.64)	0.991
Current smoking	0.74 (0.55–1.00)	0.054	0.94 (0.56–1.57)	0.824	0.94 (0.56–1.57)	0.816
Alcohol use (≥1 drinking/week)	0.64 (0.50–0.82)	<0.001	0.91 (0.65–1.26)	0.612	0.91 (0.65–1.27)	0.588
Sun exposure						
<2 hours/day	1.00 (reference)		1.00 (reference)		1.00 (reference)	
2–5 hours/day	1.08 (0.81–1.44)	0.563	0.82 (0.58–1.16)	0.274	0.82 (0.58–1.16)	0.278
>5 hours/day	1.55 (1.12–2.15)	0.007	1.04 (0.70–1.53)	0.842	1.03 (0.70–1.53)	0.847
Asthma	1.31 (0.73–2.34)	0.363	0.78 (0.38–1.61)	0.514	0.78 (0.38–1.61)	0.497
Atopic dermatitis	0.86 (0.42–1.75)	0.689	0.90 (0.38–2.12)	0.821	0.90 (0.38–2.12)	0.875
Rheumatoid arthritis	1.37 (0.73–2.60)	0.321	1.07 (0.50–2.27)	0.858	1.06 (0.49–2.27)	0.862
SBP (mmHg)	1.02 (1.01–1.03)	<0.001	1.00 (0.99–1.01)	0.904	1.00 (0.99–1.01)	0.924
DBP (mmHg)	0.99 (0.78–1.00)	0.043	0.99 (0.97–1.01)	0.400	0.99 (0.97–1.01)	0.398
Fasting plasma glucose (mg/dL)	1.01 (1.00–1.01)	0.030	1.00 (0.99–1.01)	0.511	1.00 (0.99–1.01)	0.491
Total cholesterol (mg/dL)	0.99 (0.99–1.00)	0.076	0.99 (0.99–1.00)	0.108	0.99 (0.99–1.00)	0.103
Triglyceride (mg/dL)	1.00 (0.99–1.00)	0.814	1.00 (0.99–1.00)	0.819	1.00 (0.99–1.00)	0.894
Increased total serum IgE	1.48 (1.17–1.88)	0.001	1.36 (1.00–1.85)	0.048	1.37 (1.01–1.86)	0.044
Sensitization to specific allergen						
*Dermatophagoides farina*	1.07 (0.83–1.38)	0.565	1.12 (0.82–1.53)	0.466	1.12 (0.82–1.54)	0.450
Cockroaches	1.04 (0.77–1.39)	0.804	0.86 (0.59–1.24)	0.428	0.84 (0.58–1.22)	0.387
Dogs	0.99 (0.55–1.77)	0.974	0.89 (0.45–1.76)	0.748	0.89 (0.45–1.78)	0.761

BMI, body mass index; DBP, diastolic blood pressure; IgE, immunoglobulin E; SBP, Systolic blood pressure.

*Model 1 was adjusted for age, sex, body mass index, smoking, alcohol use, and sun exposure.

^†^Model 2 was adjusted for all of the factors in Model 1 and systolic blood pressure, fasting plasma glucose, total cholesterol, asthma, atopic dermatitis, and rheumatoid arthritis.

Multivariable logistic regression was performed in each cataract subtype group to examine the association of cataracts with both total and allergen-specific serum IgE levels. The OR results are listed in Table 4. In a univariate analysis, we found that participants with increased IgE levels had a significant risk of nuclear-type cataract (OR = 1.60; *P* = 0.001). Increased IgE remained a significant risk factor for nuclear cataract after adjustment for confounding factors in Model 1 (OR = 1.41; *P* = 0.036) and Model 2 (OR = 1.43; *P* = 0.032). Sensitization to specific antigens was not a significant risk factor for any type of cataract.

**Table 4:** Odds ratios for the association of each cataract type with total and allergen-specific serum IgE.

	Increased total IgE				Sensitization to *Dermatophagoides farina*		Sensitization to Cockroaches		Sensitization to Dogs	
	OR (95% CI)	*P*-value			OR (95% CI)	*P*-value	OR (95% CI)	*P*-value	OR (95% CI)	*P-*value
Cortical cataract										
Crude	1.35 (0.87–2.08)		0.175		1.11 (0.71–1.75)	0.636	0.85 (0.49–1.50)	0.592	0.23 (0.03–1.71)	0.153
Model 1*	1.25 (0.78–2.01)		0.344		1.17 (0.72–1.89)	0.508	0.79 (0.44–1.43)	0.795	0.23 (0.03–1.75)	0.235
Model 2†	1.26 (0.78–2.03)		0.333		1.18 (0.73–1.90)	0.500	0.79 (0.43–1.43)	0.792	0.24 (0.03–1.81)	0.168
Nuclear cataract										
Crude	1.60 (1.20–2.12)		0.001		1.24 (0.93–1.67)	0.137	1.28 (0.91–1.79)	0.145	1.15 (0.59–2.24)	0.668
Model 1*	1.41 (1.02–1.94)		0.036		1.24 (0.90–1.72)	0.185	1.14 (0.78–1.65)	0.483	1.05 (0.52–2.12)	0.885
Model 2†	1.42 (1.03–1.96)		0.032		1.25 (0.90–1.74)	0.170	1.13 (0.78–1.65)	0.563	1.03 (0.51–2.08)	0.926
Anterior subcapsular cataract										
Crude	0.84 (0.18–3.79)		0.826		0.33 (0.03–2.72)	0.301	0.65 (0.07–5.49)	0.699	NA	NA
Model 1*	0.80 (0.15–4.20)		0.798		0.33 (0.03–2.93)	0.320	0.71 (0.07–6.52)	0.764	NA	NA
Model 2†	0.82 (0.15–4.37)		0.817		0.29 (0.03–2.65)	0.273	0.69 (0.07–6.65)	0.749	NA	NA
Posterior subcapsular cataract										
Crude	1.12 (0.07–18.69)		0.932	NA		NA	NA	NA	NA	NA
Model 1*	1.95 (0.10–37.21)		0.655	NA		NA	NA	NA	NA	NA
Model 2†	2.07 (0.10–41.15)		0.632	NA		NA	NA	NA	NA	NA
Mixed cataract										
Crude	0.93 (0.53–1.61)		0.797		0.56 (0.29–1.08)	0.086	0.49 (0.20–1.16)	0.107	NA	NA
Model 1*	0.76 (0.40–1.41)		0.389		0.59 (0.29–1.20)	0.149	0.41 (0.16–1.03)	0.060	NA	NA
Model 2†	0.81 (0.43–1.53)		0.522		0.53 (0.26–1.10)	0.100	0.39 (0.15–1.04)	0.055	NA	NA

NA, not assessed (due to too low prevalence of each type of cataract).

*Model 1 was adjusted for age, sex, body mass index, smoking, alcohol use, and sun exposure.

^†^Model 2 was adjusted for all of the factors on Model 1 and systolic blood pressure, fasting plasma glucose, total cholesterol, asthma, atopic dermatitis, and rheumatoid arthritis.

**Fig 3:**
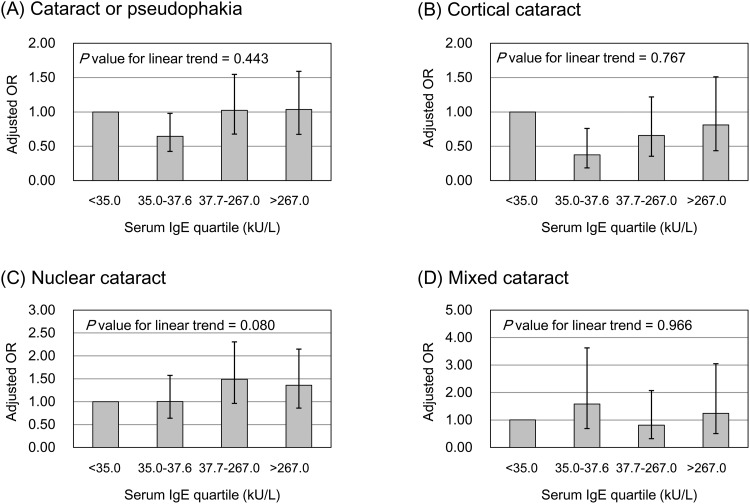
Effect of serum immunoglobulin E concentration on the odds ratios for each cataract subtype. The odds ratios of anterior and posterior subcapsular cataracts were not assessed because of a low prevalence in our study population.

To explore the more specific association between IgE levels and age-related cataracts, we carried out additional analyses involving the quartiles of IgE level. The ORs for each quartile of serum IgE concentration, adjusted for the variables in the fully adjusted model (Model 2), are shown in Fig 3. The trend analysis showed no significant linear pattern in the relationship between serum IgE concentration and the risk of any type of cataract. However, the outcome fornuclear cataract showed a weak association with serum IgE level, although it was not statistically significant. Compared with the first IgE quartile, the adjusted ORs were 1.00, 1.49, and 1.36 in the second, third, fourth quartile, respectively (*P* for linear trend = 0.080). Subgroup analyses were also performed in each age group. As shown in Table 5, an increased IgE level did not significantly affect cataract prevalence in younger age groups (40 to 59 years). However, cataract patients tended to have increased total IgE levels in older age groups (≥60 years old).

**Table 5:** The prevalence and odds ratio of cataract in patients with and without an increased total serum IgE level. Data from examined age groups are shown for comparison.

	Increased total IgE (Serum IgE ≥ 100 kU/L)	Not increased total IgE (Serum IgE < 100 kU/L)	*P*-value for χ^2^ test	Adjusted OR of increased total IgE for cataract* (95% CI)
40–49 years old				
Cataract or pseudophakia (%)	21 (5.0)	23 (5.5)	0.220	1.40 (0.72–2.71)
No cataract (%)	142 (34.1)	230 (55.3)		
50–59 years old				
Cataract or pseudophakia (%)	69 (17.7)	64 (16.4)	0.753	1.23 (0.76–2.01)
No cataract (%)	128 (32.9)	127 (32.7)		
≥60 years old				
Cataract or pseudophakia (%)	143 (39.0)	118 (32.2)	0.083	1.54 (0.93–2.53)
No cataract (%)	47 (12.8)	58 (15.8)		

*Adjusted for age, sex, body mass index, smoking, alcohol use, sun exposure, systolic blood pressure, fasting plasma glucose, total cholesterol, asthma, atopic dermatitis, and rheumatoid arthritis.

## Discussion

To our knowledge, this study was the first to evaluate the association between serum IgE levels and age-related cataracts in the general population. Our results demonstrated that study participants with increased total serum IgE levels had a significantly higher OR for age-related cataract after adjustment for confounding factors. In particular, increased IgE was significantly associated with nuclear cataract—the most common form of age-related cataract. However, neither of these findings was true of IgE sensitization to allergens in our regression models. The trend analysis of the ORs did not show a significant linear trend between the risk for age-related cataract and the serum total IgE quartiles. Since the analysis used data from a nationally representative sample of the population and was adjusted for a wide range of confounders, our results support the hypothesis that increased serum IgE levels are associated with age-related cataract.

Recently, age-related cataract has been seen as a multifactorial disease that may be related not only to age and exposure to ultraviolet, but also to systemic conditions [5]. Previous research has revealed that ocular inflammation induced by systemic risk factors—such as smoking, autoimmune diseases, and metabolic disorders—influence cataract formation [31,32]. The major biochemical explanation for this association is that inflammation in the aqueous humor leads to oxidation of the lens proteins [33]. Previous researchers have found that nitric oxide in the aqueous humor, which is induced by several cytokines, may play an important role in the development of age-related cataract [34]. Nitric oxide is a highly reactive molecule that can induce several chemical reactions that denature lens proteins [35]. In addition, one population-based study showed that several systemic inflammatory mediators (high sensitivity C-reactive protein, tumor necrosis factor α, interleukin-6, and intracellular adhesion molecule-1), were associated with age-related cataracts. The authors suggested that nuclear cataracts are related to vascular endothelial dysfunction, which is in turn associated with systemic inflammation, hypertension, and smoking [19]. Interestingly, binding of the mast cell Fc receptor to IgE produces nitric oxide and damages the vascular endothelium [36]. Therefore, these previous investigations may indicate how increased IgE leads to cataract formation.

The possible association between IgE and cataract formation maybe mediated by the mast cell in several ways: the mast cell may aggravate the oxidative status of the lens proteins directly (by activating inflammatory cascades) or indirectly (by increasing vascular permeability). Furthermore, mast cells release several inflammatory mediators, such as cytokines, chemokines, histamine, proteoglycan, and mast cell protease, following IgE stimulation [9]. These mediators are associated with all stages of the inflammatory process, including vasodilation, increase of vascular permeability, and inflammatory cell recruitment [37]. Activation of mast cell induces not only immediate hypersensitivity, but also late responses that cause long-term activation of immune cells; [38] this long-term inflammatory response may aggravate age-related changes such as oxidation of the lens protein. Moreover, a change in vascular permeability may also lead to increased concentrations of oxidative molecules such as nitric oxide in the aqueous humor [39]. These processes have been evidenced by a previous investigation reporting that lens opacity is associated with changes in the components of the aqueous humor [40].

In the present study, the adjusted multivariable regression models revealed an independent relationship between increased IgE and nuclear cataracts. In our analyses, we adjusted for potential risk factors of age-related cataract; nonetheless, our results may have been influenced by various chronic illnesses associated with both cataract formation and increased IgE level. Recent studies have revealed that IgE-mediated events can lead to several chronic diseases, including not only allergic conditions associated with hypersensitivity, but also metabolic syndrome. In fact, increased serum IgE has been associated with diabetes, hypertension, atherosclerosis, and ischemic heart disease; all these conditions are also related to cataract formation [13]. Furthermore, steroids, which are used to treat IgE-mediated and autoimmune diseases, such as asthma, atopic dermatitis, and rheumatoid arthritis, can also oxidize lens proteins [25]. However, in the current analysis, these conditions were controlled for.

Considering that increased IgE levels were associated with age-related cataracts, it was surprising that IgE sensitization to specific allergens, including *Dermatophagoides farinae*, cockroach, and dog, showed no significant association. The reason for this finding is unknown. Because sensitization to most of these allergens occurs in childhood, the progression of age-related cataracts in old-aged persons may not be closely associated with the process [27]. According to previous reports, total IgE levels in adults can be influenced by autoimmune disease or lifestyle factors [11]. For example, an association between increased total IgE and smoking has been found in the general population [41]. Additionally, although the mechanism remains unclear, chronic alcohol consumption is also associated with increased total IgE [42]. Overall, total IgE levels may play a role as an inflammatory biomarker that is closely associated with inflammatory responses to non-specific environmental factors, as well as with allergic reactions to specific allergens [13]. Therefore, it stands to reason that increased IgE levels may be associated with age-related cataracts despite the lack of association with IgE sensitization to specific allergens.

This study had certain limitations. Firstly, our analyses did not establish a causal relationship because of their cross-sectional nature. Moreover, our health data was based on a health interview survey taken on one occasion. Fasting plasma glucose, body mass index, blood pressure, and serum IgE may all differ depending on the time of measurement. Secondly, this was a single, Asia-specific study. Generally, the incidence and type of cataract are influenced by genetic background [43]. Therefore, it is uncertain whether our results are relevant to other ethnic groups. Third, slit lamp examinations were performed by many different investigators, and an inter-observer variation could have been introduced, as already discussed in a previous study that used KNHANES data [44]. Despite this limitation, examinations were adequately performed by trained investigators, and risk factors associated with cataract were identified at a nation-wide level. Fourth, dietary intake and nutritional factors were not fully considered in this study. As dietary intake data were collected using an interviewer-administered questionnaire, reporting errors may have occurred because of a recall bias. However, adjustments for plasma glucose, total cholesterol, serum vitamin D concentration, total calorie intake, total vitamin A intake, and total vitamin C intake did not alter our findings (S1 Table). Lastly, we did not consider concomitant corticosteroids, because information about inhaled and oral steroid use was not fully collected as part of the KNHANES 2010. Moreover, the 2010 KNHANES did not collect data regarding several chronic diseases associated with steroid use (e.g., allergic rhinitis [45], inflammatory bowel disease [46], and uveitis [47]); such data could have confounded our findings. In future, it will be necessary to adopt systemic approaches when investigating the long-term effects of drugs associated with cataracts.

In conclusion, our findings demonstrated that increased total IgE levels significantly associated with age-related cataracts. Specifically, increased IgE was significantly related to nuclear cataracts. Due to the aforementioned limitations, further studies involving a larger sample size and other ethnicities are still needed. In addition, future studies should clarify more specifically the role of IgE in the development of age-related cataracts.

## Supporting Information

S1 Table Further logistic regression analyses including serum vitamin D, total calorie intake, and total intake of vitamin A and C. (DOCX)
