# Supplementary material for: Age-Related Cataract Is Associated with Elevated Serum Immunoglobulin E Levels in the South Korean Population: A Cross-Sectional Study
Source: PLoS One. 2016 Nov 18;11(11):e0166331. doi: 10.1371/journal.pone.0166331 (PMC5115736; doi:10.1371/journal.pone.0166331)
Supplement: S1 Table — (DOCX) [file pone.0166331.s001.docx]

**S1 Table. Additional adjustment for nutritional factors.** Further further logistic regression analyses including serum vitamin D, total calorie intake, and total intake of vitamin A and C.

|  | Increased total IgE | |  | Sensitization to *Dermatophagoides farina* | |  | Sensitization to Cockroaches | |  | | Sensitization to Dogs | | | |  |
| --- | --- | --- | --- | --- | --- | --- | --- | --- | --- | --- | --- | --- | --- | --- | --- |
|  | OR* (95% CI) | *P*-value |  | OR* (95% CI) | *P*-value |  | OR* (95% CI) | *P*-value |  | | OR* (95% CI) | | *P-*value | |  |
| Cataract or pseudophakia | 1.38  (1.02–1.88) | 0.039 |  | 1.13  (0.82–1.55) | 0.424 |  | 0.84  (0.58–1.22) | 0.301 | |  | | 0.88  (0.45–1.77) | | 0.665 | |
| Cortical cataract | 1.28  (0.79–2.05) | 0.310 |  | 1.19  (0.73–1.91) | 0.458 |  | 0.79  (0.43–1.43) | 0.717 | |  | | 0.24  (0.03–1.81) | | 0.161 | |
| Nuclear cataract | 1.45  (1.05–1.99) | 0.023 |  | 1.24  (0.88–1.74) | 0.189 |  | 1.12  (0.78–1.63) | 0.626 | |  | | 1.03  (0.51–2.08) | | 0.862 | |
| Anterior subcapsular cataract | 0.83  (0.15–4.39) | 0.928 |  | 0.28  (0.02–2.63) | 0.249 |  | 0.68  (0.07–6.63) | 0.771 | |  | | NA | | NA | |
| Posterior subcapsular cataract | 2.10  (0.11–41.49) | 0.593 |  | NA | NA |  | NA | NA | |  | | NA | | NA | |
| Mixed cataract | 0.84  (0.46–1.57) | 0.60 |  | 0.55  (0.27–1.11) | 0.096 |  | 0.38  (0.15–1.03) | 0.062 | |  | | NA | | NA | |

*Adjusted for age, sex, body mass index, smoking, alcohol use, sun exposure, systolic blood pressure, fasting plasma glucose, total cholesterol, asthma, atopic dermatitis, rheumatoid arthritis, serum vitamin D, total calorie intake, and total intake of vitamin A and C.

Nutritional characteristics of the study participants

|  | Total participants  (N = 1170) | Cataract or pseudophakia  (N = 438) | No cataract  (N = 732) | *P*-value |
| --- | --- | --- | --- | --- |
| Serum vitamin D (ng/dL) | 18.9 ± 6.7 | 19.3 ± 7.0 | 18.5 ± 6.4 | 0.043 |
| Total calorie intake (kcal) | 2032 ± 845 | 1886 ± 777 | 2123 ± 874 | <0.001 |
| Total vitamin A intake (ug) | 886 ± 1005 | 819 ± 914 | 928 ± 1056 | 0.091 |
| Total vitamin C intake (mg) | 116 ± 92 | 112 ± 91 | 119 ± 92 | 0.254 |
